# A role of *ADAR2* and RNA editing of glutamate receptors in mood disorders and schizophrenia

**DOI:** 10.1186/1756-6606-7-5

**Published:** 2014-01-21

**Authors:** Mie Kubota-Sakashita, Kazuya Iwamoto, Miki Bundo, Tadafumi Kato

**Affiliations:** 1 Laboratory for Molecular Dynamics of Mental Disorders, RIKEN Brain Science Institute, Wako, Saitama 351-0198, Japan; 2 Department of Molecular Psychiatry, Graduate School of Medicine, The University of Tokyo, 7-3-1 Hongo, Bunkyo, Tokyo 113-8655, Japan

**Keywords:** RNA editing, Adenosine deaminase acting on RNA type 2, AMPA/kainate receptors, Serotonin 2C receptor

## Abstract

**Background:**

Pre-mRNAs of 2-amino-3-(3-hydroxy-5-methyl-isoxazol-4-yl)-propanoic acid (AMPA)/kainate glutamate receptors undergo post-transcriptional modification known as RNA editing that is mediated by adenosine deaminase acting on RNA type 2 (*ADAR2*). This modification alters the amino acid sequence and function of the receptor. Glutamatergic signaling has been suggested to have a role in mood disorders and schizophrenia, but it is unknown whether altered RNA editing of AMPA/kainate receptors has pathophysiological significance in these mental disorders. In this study, we found that *ADAR2* expression tended to be decreased in the postmortem brains of patients with schizophrenia and bipolar disorder.

**Results:**

Decreased *ADAR2* expression was significantly correlated with decreased editing of the R/G sites of AMPA receptors. In heterozygous *Adar2* knockout mice (*Adar2*^+/−^ mice), editing of the R/G sites of AMPA receptors was decreased. *Adar2*^+/−^ mice showed a tendency of increased activity in the open-field test and a tendency of resistance to immobility in the forced swimming test. They also showed enhanced amphetamine-induced hyperactivity. There was no significant difference in amphetamine-induced hyperactivity between *Adar2*^+/−^ and wild type mice after the treatment with an AMPA/kainate receptor antagonist, 2,3-dihydroxy-6-nitro-7-sulfamoyl-benzo[f]quinoxaline.

**Conclusions:**

These findings collectively suggest that altered RNA editing efficiency of AMPA receptors due to down-regulation of *ADAR2* has a possible role in the pathophysiology of mental disorders.

## Background

Mood disorders and schizophrenia are major psychiatric diseases that cause severe psychosocial impairment. Because many antidepressant and antipsychotic drugs act on the serotonin transporter and serotonin receptors, the serotonin has been implicated as having a role in these diseases [1]. However, among the drugs acting on glutamate receptors, phencyclidine causes schizophrenia-like psychosis [2] and ketamine has rapid antidepressive efficacy [3]. These findings suggest a possible role of altered glutamatergic neurotransmission in mood disorders and schizophrenia [1].

To date, numerous studies using genome analysis, positron emission tomography, and postmortem brain analysis have revealed possible roles of serotonergic and glutamatergic pathways in schizophrenia and mood disorders [1]. However, the detailed molecular mechanisms of these diseases have yet to be totally elucidated. Among the receptors in these pathways, pre-mRNAs of the serotonin 2C receptor (*HTR2C*) and two classes of ionotropic glutamate receptors, 2-amino-3-(3-hydroxy-5-methyl-isoxazol-4-yl)-propanoic acid (AMPA) and kainate receptors, undergo RNA editing [4,5].

RNA editing is a post-transcriptional modification of pre-mRNA, which is mediated by adenosine deaminases acting on RNA (ADAR) enzymes. Research has especially focused on adenosine-to-inosine (A-to-I) editing of *HTR2C* and AMPA/kainate receptors by ADAR2 [6]. *HTR2C* undergoes editing at five sites (from A to E), which results in amino acid changes and causes functional alteration. Among the editing sites of AMPA/kainate receptors, two sites (Q/R and R/G) that result in amino acid changes and have functional significance, have been relatively well studied. The Q/R site is almost 100% edited in *GRIA2*, and loss of editing at this site causes enhanced Ca^2+^ permeability, resulting in cellular dysfunction [6]. The R/G site is not fully edited, which changes the kinetics of desensitization [7].

Numerous studies have investigated altered RNA editing of *HTR2C* in postmortem brains of patients with mental disorders, but the findings have not been consistent. While several studies did not show any specific alteration of RNA editing in mental disorders [8-11], other studies showed disease-specific alteration, such as increased D site editing in depression and increased A site editing in suicide completers [12], decreased B site editing in schizophrenia [13], and increased E site and decreased D site editing in depression [14]. Among these studies, increased A site editing in suicide completers has been shown to be relatively consistent [9,10,12,15]. A recent study using a next-generation sequencer [11] showed no robust alteration of RNA editing of *HTR2C* in schizophrenia and depression, except that a trend of decreased editing at the C, D, and E sites in nonsuicidal depression was found. However, a trend of increased A site editing in depressive patients that died by suicide is compatible with previous studies.

The number of studies regarding the RNA editing of AMPA/kainate receptors is relatively small. While several studies showed no alteration in schizophrenia [16,17] and bipolar disorder [17], a recent study revealed altered editing of the I/V site of *GRIK2* in bipolar disorder [6]. The role of RNA editing of glutamate receptors has drawn attention because the glutamatergic hypothesis of mood disorders has recently been established [18].

As already mentioned, there is no consensus on what kind of alteration of RNA editing is characteristic of schizophrenia and mood disorders. Possible reasons for such discordance include the effect of confounding factors in postmortem brain studies, such as medication and premortem or postmortem changes, and complex interactions between the cause of death (such as suicide) and mental disorders. Recently, Lyddon and colleagues argued that there are two factors involved in the altered editing of *HTR2C* in mood disorders; one is decreased *ADAR2* expression associated with decreased RNA editing of *HTR2C*, and the other is increased A site editing in suicide [15].

Collectively, it is difficult to elucidate how RNA editing is related to mental disorders by postmortem brain analysis alone. To understand the pathophysiological significance of altered *ADAR2* expression and RNA editing in mental disorders, integration of human postmortem brain analysis and animal model studies is crucial. In this study, we investigated the possible roles of altered *ADAR2* expression and RNA editing of AMPA/kainate glutamate receptors through an analysis of these factors in human postmortem brains. Molecular, behavioral, and pharmacological analyses of heterozygous *Adar2* knockout mice (*Adar2*^+/−^ mice) were also conducted to elucidate the roles of RNA editing in schizophrenia and mood disorders.

## Results

### Overview

We analyzed the gene expression levels of the *ADAR2* and RNA editing status of AMPA/kainite glutamate receptors in two sets of postmortem human brain samples donated by the Stanley Medical Research Institute, Array Collection and Neuropathology Consortium (Table 1). To elucidate the molecular neurobehavioral consequence of reduced *ADAR2* expression, we analyzed the RNA editing status of *Htr2c* and AMPA/kainate glutamate receptors in *Adar2*^+/−^ mice; the behavior of the mice was analyzed by a comprehensive battery of behavioral tests. To further understand the molecular basis of the hyperactivity of the mice, pharmacological experiments using amphetamine and the selective AMPA receptor antagonist, 2,3-dihydroxy-6-nitro-7-sulfamoyl-benzo[f]quinoxaline (NBQX) were performed.

**Table 1:** Subjects for RNA editing and expression analysis of ADARs

	**Bipolar disorder**	**Schizophrenia**	**Control**	**Depression**
**Array Collection (all samples)**				
n	32	35	34	NA
Sex (F:M)	17:15	9:26	9:25	
Age^a)^	45.6 ± 11.0	42.6 ± 8.5	44.1 ± 7.7	
PMI^b)^	36.3 ± 17.9	31.4 ± 15.5	29.6 ± 13.0	
Brain pH^c)^	6.43 ± 0.30^g)^	6.47 ± 0.24^h)^	6.60 ± 0.27	
**Array Collection (pH-adjusted)**				
n	19	24	29	NA
Sex (F:M)	11:8	9:15	6:23	
Age^d)^	46.1 ± 9.9	42.6 ± 8.5	44.6 ± 7.7	
PMI^e)^	39.9 ± 20.2	35.0 ± 14.8	30.2 ± 12.5	
Brain pH^f)^	6.63 ± 0.15	6.61 ± 0.14	6.69 ± 0.17	
**Neuropathology Consortium Samples**^i)^				
n	11	13	14	11
Sex (F:M)	3:8	5:8	5:9	5:6
Age	39.4 ± 12.4	43.5 ± 13.6	49.0 ± 10.4	46.3 ± 10.5
PMI	31.5 ± 15.5	33.0 ± 14.9	22.6 ± 9.2	27.0 ± 11.9
Brain pH	6.25 ± 0.20	6.15 ± 0.25	6.30 ± 0.21	6.18 ± 0.24

NA, not available.

^a)^One way ANOVA, *F* = 0.94, *P* = 0.39, ^b)^*F* = 1.61, *P* = 0.20, ^c)^*F* = 3.5, *P* = 0.031.

^d)^One-way ANOVA, *F* = 0.89, *P* = 0.41, ^e)^One-way ANOVA, *F* = 2.2, *P* = 0.11.

^f)^One-way ANOVA, *F* = 1.7, *P* = 0.18, ^g)^*t* = -2.4, *P* = 0.017 to control.

^h)^*t* = 2.0, *P* = 0.042 to control.

^i)^No significant difference among 4 groups were found for age, PMI, brain pH by One-way ANOVA.

### *ADAR2* expression in human postmortem brains

In the Array Collection samples, there was a significant difference in the *CFL1* (*cofilin 1)*-normalized *ADAR2* expression level between diagnoses [one-way analysis of variance (ANOVA), *P* < 0.05]. Patients with schizophrenia showed significantly lower *ADAR2* expressions in both Glyceraldehyde 3-phosphate dehydrogenase (*GAPDH)-* and *CFL1*-normalized data (*P* < 0.05). Patients with bipolar disorder also showed a nonsignificant trend in the same direction, and it was close to significance (*P* = 0.05) for the *CFL1*-normalized *ADAR2* expression level (Table 2). There was a significant correlation between brain pH and *ADAR2* expression (*GAPDH*-normalized *ADAR2*: *r* = 0.28, *P* = 0.001; *CFL1*-normalized *ADAR2*: *r* = 0.222, *P* < 0.05). Because low pH in brain affected the measurement of postmortem brain gene expression [19], we selected samples with high brain pH (pH 6.4 or higher). This threshold was determined in our previous gene expression study [20]. We found that the results were similar after selection of high pH samples (Table 2). A similar nonsignificant trend for decreased *ADAR2* expression was found in the Neuropathology Consortium samples (Table 2). There was no significant difference of *ADAR1* expression between diagnoses (Table 2).

**Table 2:** Expression levels of ADARs in postmortem brains of patients with mental disorders

**Array collection samples**				
	All samples (normalized by *GAPDH*)		All samples (normalized by *CFL1*)	
Diagnosis	*ADAR1*	*ADAR2*	*ADAR1*	*ADAR2*
Bipolar disorder	0.0219 ± 0.0090	0.0025 ± 0.0011	0.0397 ± 0.0163	0.0046 ± 0.0018^b)^
Schizophrenia	0.0216 ± 0.0086	0.0024 ± 0.0008^a)^	0.0393 ± 0.0192	0.0043 ± 0.0015^c)^
Control	0.0232 ± 0.0063	0.0029 ± 0.0009	0.0477 ± 0.0380	0.0054 ± 0.0015
One-way ANOVA	NS	*F =* 2.11*, P =* 0.120	NS	*F* = 3.86, *P* = 0.024
	pH-adjusted (normalized by *GAPDH*)		pH-adjusted (normalized by *CFL1*)	
Diagnosis	*ADAR1*	*ADAR2*	*ADAR1*	*ADAR2*
Bipolar disorder	0.0028 ± 0.0010	0.0244 ± 0.0096	0.0050 ± 0.0016	0.0442 ± 0.0180
Schizophrenia	0.0025 ± 0.0008^d)^	0.0231 ± 0.0088	0.0043 ± 0.0013^e)^	0.0401 ± 0.0149
Control	0.0031 ± 0.0008	0.0238 ± 0.0054	0.0054 ± 0.0014	0.0420 ± 0.0080
One-way ANOVA	*F* = 3.09*, P* = 0.052	NS	*F* = 4.37*, P* = 0.016	NS
**Neuropathology Consortium Samples**				
	Normalized by *GAPDH*		Normalized by *CFL1*	
Diagnosis	*ADAR1*	*ADAR2*	*ADAR1*	*ADAR2*
Bipolar disorder	0.0025 ± 0.0016^f)^	0.0110 ± 0.0071	0.0122 ± 0.0097	0.0026 ± 0.0017^g)^
Depression	0.0030 ± 0.0012	0.0107 ± 0.0036	0.0043 ± 0.0013^e)^	0.0039 ± 0.0012
Schizophrenia	0.0029 ± 0.0006	0.0107 ± 0.0045	0.0151 ± 0.0051	0.0039 ± 0.0010
Control	0.0033 ± 0.0008	0.0119 ± 0.0054	0.0133 ± 0.0062	0.0037 ± 0.0010
One-way ANOVA	NS	NS	NS	*F = 5.6, P = 0.008*

Values are given in ± mean SD.

^a)^*t* = 2.22, *P =* 0.029 to control, ^b)^*t =* -1.92, *P =* 0.05 to control.

^c)^*t =* 2.90, *P =* 0.005 to control, ^d)^*t =* -2.65, *P =* 0.01, ^e)^*t =* -3.12, *P =* 0.003.

^f)^*t =* -1.70, *P =* 0.09 to control, ^g)^*t =* -2.0, *P =* 0.06 to control.

NS: not significant.

For one of 13 subjects with schizophrenia in Neuropathology Consortium, data was missing.

### RNA editing changes in *Adar2*^+/−^ mice

The initial report showed that homozygous *Adar2* knockout is lethal in mice due to seizures caused by a marked decrease of the Q/R site of *Gria2*, which results in enhanced Ca^2+^ permeability of the AMPA receptors [21]. *Adar2*^+/−^ mice showed less prominent alteration of RNA editing and were viable. Thus, *Adar2*^+/−^ might be a better model for mental disorders.

**Figure 1:**
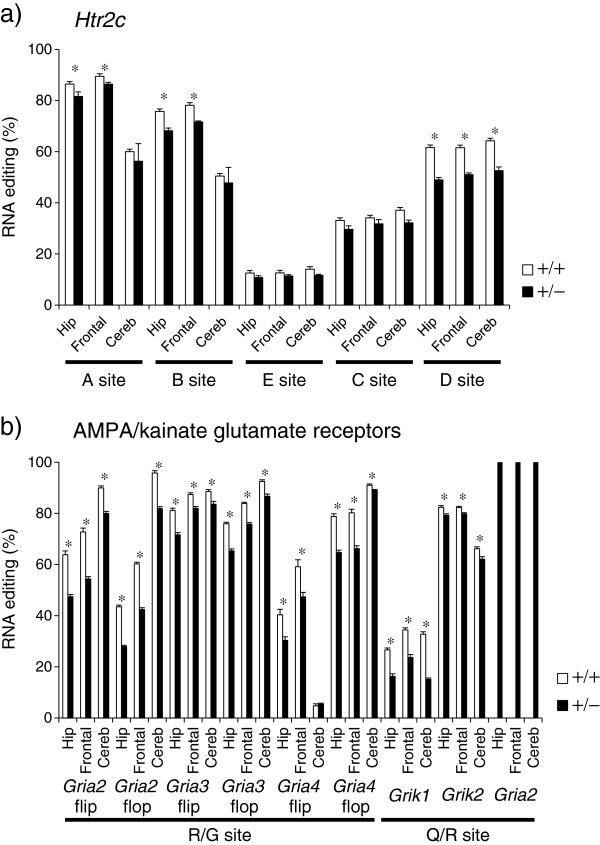
**RNA editing of *Htr2c *and AMPA/kainate glutamate receptors in *Adar2***^**+/− **^**mice. a) ***Htr2c*. **b)** AMPA/kainate glutamate receptors. *P* < 0.05 by Student *t*-test. Hip denotes hippocampus, Frontal denotes frontal cortex, and Cereb denotes cerebellum. White bars indicate the WT mice, black bars indicate the *Adar2*^+/−^ mice. Data represent mean and error bars indicated by standard errors (n = 10 for each genotype).

We first characterized the effect of *Adar2*^+/−^ on RNA editing status. Examination of *Htr2c* editing revealed that the E and C sites were not altered, whereas the A, B, and D sites had significantly decreased editing efficiency, except in the cerebellum, where only the D site showed significant alteration (Figure 1a). The alteration of the A and B sites was relatively modest (10% or less), whereas the change in the D site was larger (up to 20%).

With regard to the AMPA/kainate receptors, the Q/R site of *Gria2* was almost fully edited (Figure 1b). Editing of the Q/R sites of *Grik1* and *Grik2* was slightly decreased. There are two alternative spliced isoforms of flip and flop types that have different desensitization kinetics. Because the R/G site is located just before this alternative spliced exon [7], the R/G sites were separately examined for two alternative spliced isoforms. We found that RNA editing of the R/G site was generally decreased in both the flip and flop isoforms for all receptors investigated (*Gria2*, *Gria3*, and *Gria4*). The R/G site showed a 10–36% decrease except for *Gria4* in the cerebellum, where minimal or no alteration in editing efficiency was observed.

### RNA editing in human postmortem brains

**Figure 2:**
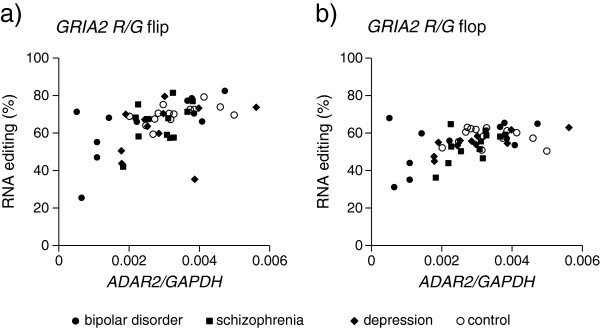
**RNA editing of *GRIA2 *R/G site and *ADAR2 *expression level in human postmortem brains.** Relationship between expression level of *ADAR2* and editing efficacy at R/G sites of *GRIA2*. **a) ***GRIA2*, flip isoform. **b) ***GRIA2*, flop isoform. The vertical axis means the RNA editing efficiency at the R/G site of *GRIA2*. The horizontal axis shows the mRNA expression of *ADAR2* shown by the *ADAR2/GAPDH* ratio. Each dot represents the one person’s editing efficacy at the R/G site of *GRIA2* versus their expression level of *ADAR2*. Closed circles represent bipolar disorder (n = 11), closed squares represent schizophrenia (n = 13), closed diamonds represent depression (n = 11), and open circles represent controls (n = 14). Correlation coefficients were *r* = 0.53 (*P* < 0.001) for the flip isoform and *r* = 0.42 (*P* < 0.005) for the flop isoform. The information of each subject was shown in Neuropathology Consortium samples of Table 1.

We previously reported RNA editing efficiency of *HTR2C* in the Consortium samples, which showed an increase at the D site in depression and an increase at the A site in suicide completers [12]. In this study, we measured the editing efficiency of AMPA/kainate receptors in the same sample set. As expected, the Q/R sites were almost fully edited in all groups. There was no significant alteration in RNA editing of R/G sites, but there was a tendency toward decreased editing efficiency in mood disorders (Table 3). The editing efficiency at the R/G site was significantly correlated with the *ADAR2* expression level for all transcripts investigated (*r* = 0.30–0.64, *P* < 0.05 for *GAPDH*-normalized and *r* = 0.25–0.64, *P* < 0.05 for *CFL1*-normalized data) (Table 3); however, the Q/R site of *GRIA2* was not (*P* > 0.05). As shown in Figure 2, some of the patients showed a prominent decrease in both editing efficiency of R/G sites and *ADAR2* expression levels.

**Table 3:** RNA editing of AMPA receptors in the postmortem brains of patients with mental disorders

	**R/G sites**						**Q/R sites**
	***GRIA2 *flip**	***GRIA2 *flop**	***GRIA3 *flip**	***GRIA3 *flop**	***GRIA4 *flip**	***GRIA4 *flop**	***GRIA2***
Bipolar disorder	64.4 ± 16.4	54.4 ± 12.5	66.3 ± 9.5	79.4 ± 10.6	56.0 ± 17.1	64.8 ± 15.8	96.6 ± 3.4
Depression	62.5 ± 13.8	54.9 ± 5.3	61.9 ± 9.6	82.6 ± 7.7	55.0 ± 15.8	69.9 ± 10.7	97.6 ± 3.0
Schizophrenia	66.0 ± 10.8	53.7 ± 8.0	66.0 ± 5.9	83.5 ± 9.4	60.5 ± 9.7	71.5 ± 6.1	99.0 ± 1.9
Control	70.1 ± 4.8	58.1 ± 4.4	65.0 ± 12.1	85.5 ± 3.5	63.2 ± 9.5	71.2 ± 7.5	98.8 ± 2.3
r* (*GAPDH*)	0.508	0.476	0.300	0.644	0.527	0.565	ns
r* (*CFL1*)	0.477	0.430	0.251	0.642	0.509	0.600	ns

*r = Correlation coefficient between editing efficiency of AMPA receptors and *ADAR2* expression level.

### Behavioral analysis of *Adar2*^+/−^ mice

**Figure 3:**
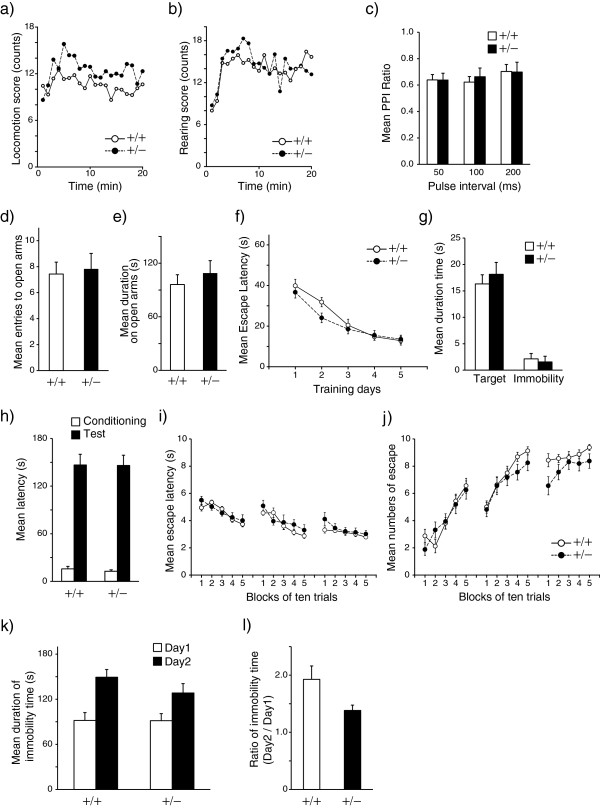
**Behavioral battery in *Adar2***^**+/− **^**mice. a,b)** Open-field test. The vertical axis is the counts measured by the interruption of infrared beams. **a)** Locomotor activity. **b)** Rearing. Closed circles indicate the *Adar2*^+/−^ mice (+/−). Open circles indicate the WT mice (+/+). No significant genotype × time interaction was found for locomotor activity. Locomotor count tended to be higher in the *Adar2*^+/−^ mice than that in the WT mice (*t* = 2.0, *P* = 0.05). RMANOVA with the intrasubject factor of time (1–20 min, df = 19) and the intersubject factor of genotype showed no significant effect of genotype or genotype × time interaction in rearing count. **c)** PPI test. There was no significant difference between genotypes. **d,e)** Elevated plus maze test. **d)** Number of entries to open arms. **e)** Time spent on open arms. There was no significant difference in the number of entries and time spent on open arms between genotypes. **f,g)** Morris water maze test. **f)** Escape latency. No significant effect of genotype was found for the escape latency by RMANOVA. **g)** Probe test. There was no significant difference between genotypes. **h)** Passive avoidance test. There was no significant effect of genotypes by two-way ANOVA. **i,j)** Active avoidance test. There was no significant interaction between time (*df* = 14) and genotype (*df* = 1) in the avoidance latency **(i)** or number of avoidances **(j)** by RMANOVA (*P* = 0.05). **k,l)** Forced swimming test. **k)** Duration of immobility. **l)** The ratio of immobility time on the second day to the first day. Data represent mean and error bars indicated by standard errors (n = 16 for each genotype).

The present findings suggest that *ADAR2* is down-regulated in schizophrenia and bipolar disorder, which correlates with decreased R/G site editing of AMPA receptors. Thus, decreased *ADAR2* levels and the resultant alteration of editing efficiency at the R/G sites of AMPA receptors might have some pathophysiological significance in mood disorders and schizophrenia. To test this, we applied a battery of conventional behavioral tests to *Adar2*^+/−^ mice. The results are summarized in Figure 3. In open-field test, RMANOVA with the intrasubject factor of time (1–20 min, *df* = 19) and the intersubject factor of genotype showed a trend-level effect of genotype (*F* = 4.0, *P* = 0.054) for locomotor activity. The *Adar2*^+/−^ mice tended to be more hyperactive than the wild-type (WT) mice (Figure 3a, *t* = 2.01, *P* = 0.05). No significant difference was found in the rearing scores (Figure 3b). There was no alteration in prepulse inhibition (Figure 3c), which does not support the hypothesis that *Adar2*^+/−^ mice show schizophrenia-like sensorimotor gating abnormality. Among the factors affecting the results of the open-field test, a possible effect of anxiety was not supported because there was no significant alteration by the elevated plus maze (Figure 3d, 3e). The Morris water maze test did not show any difference, indicating that there was no marked impairment in spatial memory (Figure 3f, 3g). No significant alteration in passive avoidance test (Figure 3h) is in accordance with lack of significant alteration in elevated plus maze test. In the active avoidance test, there was no significant time × genotype interaction in the avoidance latency or number of avoidances by repeated measures (RM) ANOVA (RMANOVA) (Figure 3i, 3j). In the forced swimming test (Figure 3k, 3l), the ratio of the immobility time on the second day to that of the first day, which is an indicator for behavioral despair, tended to be smaller in the *Adar2*^+/−^ mice (*t* = 1.9, *P* = 0.06). This suggests that *Adar2*^+/−^ mice showed a tendency of resistance to behavioral despair.

### Pharmacological experiments

**Figure 4:**
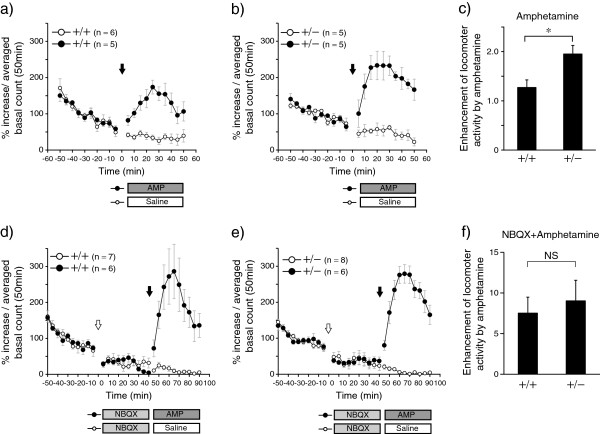
**Effects of amphetamine with or without NBQX pre-treatment on locomotor activity in *Adar2***^**+/− **^**mice. a,b)** Effect of amphetamine on locomotor activity in the WT mice **(a)** and the *Adar2*^+/−^ mice **(b)**. The horizontal axis indicates the time before and after administration of amphetamine or saline. The vertical axis indicates the activity level. Each dot represents the averaged activity level during the 5-min period. Closed circles indicate amphetamine-injected group and open circles indicate saline-injected group. Arrow indicates the time of drug administration. **c)** The vertical axis indicates the fold change of the averaged activity level during 50 min after the administration of amphetamine to the averaged activity during 50 min before the treatment. *P = 0.05 by Student *t*-test. **d,e)** Effect of NBQX on amphetamine-induced hyperactivity in the WT mice **(d)** and the *Adar2*^+/−^ mice **(e)**. The horizontal axis indicates the time before and after the administration of amphetamine or saline. The vertical axis indicates the activity level. Each dot represents the averaged activity level during the 5-min period. Closed circles indicate the amphetamine injected group and open circles indicate the saline-injected group. Arrows indicate the time of drug administration. The open arrow indicates the administration of NBQX and the closed arrow indicates the injection of amphetamine or saline. **f)** The vertical axis indicates the fold change of the averaged activity level during 50 min after the administration of amphetamine to the averaged activity during the middle 50 min after the NBQX treatment. No significant difference was found between genotypes. Data represent mean and error bars indicated by standard errors.

To further elucidate the mechanism of increased locomotor activity in *Adar2*^+/−^ mice, we examined the effect of amphetamine administration in the mice. Amphetamine is known to evoke a delayed overflow of glutamate in the brain in addition to having an impact on the dopaminergic system. It causes acute hyperactivity and behavioral sensitization [22,23]. Less editing in the GluR2 receptors would result in high Ca^2+^ permeability in AMPA receptors, which may enhance glutamate transmission in *Adar2*^+/−^ mice. Previously, it was shown that the AMPA receptor antagonist NBQX by itself had no effect on locomotor activity but prevented hyperactivity after treatment with amphetamine [24,25]. In accordance with previous studies, the locomotor activity gradually declined in two groups with treatment of saline or NBQX in both the WT and the *Adar2*^+/−^ mice in our experiment. There was no significant difference of activity level between saline injection and NBQX injection both in the WT mice and the *Adar2*^+/−^ mice [see Additional file 1: Figure S1]. Thus, we examined this behavioral trait in relation to amphetamine treatment of *Adar2*^+/−^ mice. The amphetamine treatment enhanced the activity level in both the WT and the *Adar2*^+/−^ mice (Figure 4a, 4b). RMANOVA showed significant effects of genotype (*df* = 1, *F* = 17.8, *P* <0.005) and drug (*df* = 1, *F* = 70.9, *P* < 0.001), as well as a significant interaction of genotype × drug (*df* = 1, *F* = 7.1, *P* < 0.05). The enhancement was significantly larger in the *Adar2*^+/−^ mice than in the WT mice (Figure 4c, *t* = 3.07, *P* = 0.015). Amphetamine treatment after the NBQX treatment also enhanced the locomotor activity in the *Adar2*^+/−^ mice (9.03 ± 7.16) and the WT mice (7.51 ± 5.19) (Figure 4d, 4e). RMANOVA showed significant effects of drug (*df* = 1, *F* = 58.1, *P* < 0.001) but no significant effect of genotype (*df* = 1, *F* = 0.37, *P=0.54*) and no significant interaction of genotype × drug (*df* = 1, *F* = 0.29, *P* = 0.59). There was no significant difference in the enhancement between genotypes (Figure 4f, *t* = 0.146, *P* = 0.56).

## Discussion

In this study, we performed an integrated analysis of *ADAR2* and RNA editing of AMPA/kainate glutamate receptors in human postmortem brains and model mice. Recent expression studies using postmortem brains showed no significant alteration of *ADAR2*[6,26] and significant down-regulation of *ADAR2* in major depressive disorder [15]. In our study, although the results were not consistent between two independent sample sets, we observed general down-regulation of *ADAR2* in the brains of patients with schizophrenia or mood disorders. The discrepancy in the results across studies may be partly due to the complexity of *ADAR2* transcripts [27]. In this study, we examined the gene expression level of *ADAR2* transcripts containing exons 11 and 12. Another notable feature was that some patients, but not all patients, showed drastic down-regulation of *ADAR2*, which was accompanied by decreased RNA editing at the R/G sites.

To model the down-regulation of *ADAR2* in patients with mental disorders, we analyzed *Adar2*^+/−^ mice. Altered RNA editing of *Htr2c* was characterized by a relatively large decrease at the D site with smaller changes at the A and B sites. A decrease of D site editing in depression has been reported in some [11,14], but not all [9,10,12,15] studies. The altered RNA editing of AMPA/kainate receptors in *Adar2*^+/−^ mice was characterized by preserved Q/R site editing of *Gria2* and decreased editing of R/G sites. This is consistent with a previous study using semiquantitative analysis of sequence chromatograms [21]. Although it is a limitation of the study that we did not measure the protein levels of Adar2, the alteration of RNA editing status might suggests that haploinsufficiency of *Adar2* would cause a decrease of Adar2 protein level and subsequently reduced Adar2 activity in *Adar2*^+/−^ mice [21]. Because there was some residual Q/R site editing of *Gria2* in homozygous *Adar2* knockout mice, *Adar1* might also play some role in the editing of this site as well as RNA editing of the R/G site of AMPA receptors and *Htr2c*. However, *Adar1* was not upregulated in homozygous *Adar2* knockout mice, suggesting that compensatory upregulation of *Adar1* do not play a major role in RNA editing changes in *Adar2*^+/−^ mice.

In the postmortem brains of patients with schizophrenia and mood disorders, statistically significant alteration of RNA editing of AMPA/kainate receptors was not found. The lack of prominent alteration of the Q/R site in schizophrenia is consistent with the pioneering work of Akbarian and colleagues [16]. However, there was a significant correlation between *ADAR2* expression and R/G site editing. This suggests that decreased *ADAR2* expression in patients with mental disorders has some functional impact on RNA editing, similar to that observed in *Adar2*^+/−^ mice. Indeed, the editing of R/G sites tended to be decreased in mood disorders as a whole (Table 3), and some patients showed markedly decreased editing efficiency (Figure 2). Thus, down-regulation of *ADAR2* and the resultant decreased editing of R/G sites might have some pathophysiological significance, at least in a subgroup of patients. The causes of downregulation of *ADAR2* in these disorders are unknown. A recent study searched for proteins regulating *ADAR2* and found three RNA binding proteins, RPS14, SFRS9 and DDX15 [28]. We previously performed gene ontology analysis of differentially expressed genes in the postmortem brains of patients with bipolar disorder and found that genes related to RNA binding and RNA splicing were significantly enriched [29]. Indeed, *SFRS9*, a splicing factor, was downregulated in the postmortem brain of patients with schizophrenia and bipolar disorder (unpublished finding). Thus, RNA machinery might be somehow dysregulated in bipolar disorder and schizophrenia.

Lyddon and colleagues examined the RNA editing in AMPA/kainate receptors in the same samples (i.e., the Neuropathology Consortium samples) [17]. Their finding that the flop isoform of *GRIA3* and *GRIA4* showed higher R/G site editing compared with the flip isoform was replicated in this study. They also reported that the diagnosis did not affect the RNA editing efficiency, which is compatible with the present finding that there is no significant difference between diagnoses.

In this study, *Adar2*^+/−^ mice showed slight tendencies of altered behavior by comprehensive behavioral battery. The *Adar2*^+/−^ mice showed hyperactivity in the open-field test, but they did not show altered prepulse inhibition, a candidate endophenotype in schizophrenia.

Amphetamine-induced hyperactivity was significantly enhanced in the *Adar2*^+/−^ mice. However, the difference in this enhancement was no longer significant after the pretreatment with NBQX, an inhibitor of the AMPA/kainate receptor. This suggests that the enhanced response to amphetamine seen in *Adar2*^+/−^ mice might be mediated by the enhanced glutamatergic signaling caused by altered function of AMPA receptors due to the decreased RNA editing of these receptors. However, a possibility that NBQX enhanced amphetamine-induced hyperactivity in WT mice but not in *Adar2*^+/−^ mice cannot be totally ruled out, because we did not set a control group to examine the effect of amphetamine after saline injection. Moreover, editing of *Htr2c* was also affected by haploinsufficiency of *Adar2*, and its contribution to the enhanced response cannot be ruled out.

In addition to the *HTR2C* and AMPA/kainate receptors, recent studies using next-generation sequencers showed numerous previously unidentified editing sites [30-34], although some of the initial findings could contain false-positive sites [35,36]. In spite of the controversy surrounding the bioinformatics analysis of RNA–DNA differences, many other A-to-I editing sites such as *Cyfip2*, *Kcna1*, *Blcap*, *Gabra3*, *Flna*, *Flnb*, and *Cadps*, have been experimentally validated as target editing sites of *Adar2*[37]. Thus, alteration of RNA editing of transcripts other than those for glutamate receptors and *HTR2C* can also contribute to the phenotypes observed in this study.

Horsch and colleagues performed behavioral analysis of homozygous *Adar2* knockout mice under the background of homozygous knock-in alleles of an edited version of *Gria2* to rescue severe phenotypes due to loss of Q/R site editing of *Gria2*[37]. These mice had dramatically decreased R/G site editing of *Gria2* (15%) and *Gria4* (10%) as well as profound alterations in RNA editing of *Htr2c*[21], and showed increased passive rotation in a rotarod test, impaired hearing ability, increased raring in open field test and impaired prepulse inhibition. Some of these findings might be attributable to decreased editing of *Htr2c. Adar2*^+/−^ mice did not show impairment in hearing ability and prepulse inhibition or altered response to open field test, possibly because of milder impairment in RNA editing of *Htr2c* and/or R/G site of AMPA receptors. However, regarding the open-field test, they showed increased rearing during the first 5 min but did not show any alteration in locomotor activity [37]. This difference may be due to different methodologies; however, less extensive editing abnormality in heterozygous knockout might cause a different phenotype.

Another group generated transgenic mice expressing *Adar2*[38] and found that these mice showed increased immobility time and decreased activity in the open-field test. Pairing this information with our findings that *Adar2*^+/−^ mice showed a tendency of increased activity in the open-field test and a tendency of resistance to immobility in the forced swimming test, we can postulate that the levels of *Adar2* and the resultant RNA editing changes might be related to the activity level or liability to the behavioral despair exhibited in the forced swimming test. In the *Adar2* transgenic mice, editing of the A, C, D, and E sites of *Htr2c* was also increased [39]. Together with the decreased editing of A, B, and D sites in *Adar2*^+/−^ mice, it is possible that altered A and D site editing of *Htr2c* might also contribute to the behavioral phenotypes of *Adar2*^+/−^ mice.

At this stage, we should be cautious about directly connecting the behavioral features of mice such as activity in open-field tests, forced swimming tests, and enhanced amphetamine response to mental disorders such as schizophrenia, depression, or mania. To extend this preliminary finding, other animal models and effects of psychotropic medications should also be examined. In spite of its limitations, the present study suggests that an altered expression level of *Adar2* due to haploinsufficiency affects the behavior of mice at least partly through the altered RNA editing efficiency of AMPA/kainate receptors.

## Conclusion

In conclusion, *ADAR2* expression is decreased in the postmortem brains of patients with schizophrenia and bipolar disorder, and decreased *ADAR2* expression is correlated with decreased RNA editing of the R/G site in AMPA glutamate receptors. *Adar2*^+/−^ mice showed decreased RNA editing of the R/G site of AMPA receptors. These mice showed slight behavioral changes such as hyperlocomotion in the open-field test, attenuated immobility response to the forced swimming test, and enhanced response to amphetamine. The difference of amphetamine response was not seen after the treatment with the AMPA/kainate receptor antagonist, NBQX. These findings collectively suggest a possible role of altered RNA editing efficiency of AMPA receptors due to down-regulation of *ADAR2* in the pathophysiology of mental disorders.

## Methods

### Postmortem brains

Two sets of postmortem brain samples donated by the Stanley Medical Research Institute were used for this study (https://www.stanleygenomics.org) [40]. One set was the Array Collection, consisting of 104 RNA samples extracted from the prefrontal cortex (Brodmann area 46) (34 bipolar disorder, 35 schizophrenia and 35 controls). The other set comprised frozen brain tissue samples (Brodmann area 10) from the Neuropathology Consortium. They were derived from patients with bipolar disorder (n = 15), major depression (n = 15), and schizophrenia (n = 15) and 15 controls. Diagnoses had been made according to the Diagnostic and Statistical Manual of Mental Disorders, Fourth Edition [41]. Because of the RNA quality obtained, 101 and 49 samples from the Array Collection and Neuropathology Consortium, respectively, could be used for this study (Table 1). Subjects’ demographic information is shown in Table 1. This study was approved by the Ethics Committee of RIKEN.

### Animals

*Adar2* knockout mice were developed by Higuchi and colleagues as described [21]. In brief, a targeting vector to replace exon 4 of *Adar2* with a PGK–neo gene was used for generation of a targeted embryonic stem cell line. Chimeric mice were generated by injection of this clone into C57BL/6-derived blastocysts, and homozygous *Adar2* knockout mice were bred thereafter. Genotyping of the mice was performed as described [21].

The mice were maintained in a 12-h light/12-h dark cycle. All animal experiments were approved by the local animal experiment committees of RIKEN and the Behavioral and Medical Sciences Research Consortium (BMSRC) (Akashi, Japan). Animal experiments were carried out in accordance with the National Institutes of Health Guide for the Care and Use of Laboratory Animals. All efforts were made to minimize the number of animals used and their suffering.

### Real-time quantitative reverse transcription polymerase chain reaction analysis in human brain samples

Three to five micrograms of total RNA was used for cDNA synthesis by oligo (dT) and SuperScript II reverse transcriptase (Invitrogen, Carlsbad, CA). Reverse transcription polymerase chain reaction (RT-PCR) using SYBER/GREEN I (Applied Biosystems, Foster City, CA) was performed with an ABI PRISM 7900HT (Applied Biosystems). The comparative Ct method was used for quantification according to the manufacture’s protocol (Applied Biosystems). Measurement of delta Ct was done at least in triplicate. Amplification of the single product was confirmed by monitoring the dissociation curve and by gel electrophoresis. We used two control genes (*GAPDH* and *CFL1*) for normalization. The validity of the use of *CFL1* as an internal control gene in postmortem brain samples has been shown previously [20]. Primer sequences used for the measurement of *ADAR2* are shown in Additional file 2: Table S1.

### RNA editing analysis

RNA editing levels of *GRIA2*, *GRIA3*, and *GRIA4* were determined in human samples. In the case of mice, those of *Gria2*, *Gria3*, *Gria4*, *Grik1*, and *Grik2* as well as *Htr2c* were determined. The primers used for this assay are listed in Additional file 2: Table S1. Because of the limited amount of RNA samples, RNA editing analysis in human brain was performed only on the Neuropathology Consortium samples by the primer extension combined with denaturing high-performance liquid chromatography (PE-DHPLC) method, according to a previous report [42]. In brief, after RT-PCR, extension of the primer was performed before the editing site, and it was terminated by incorporation of ddNTPs. The reaction mixture was separated and quantified by denaturing HPLC using a WAVE DNA fragment analysis system with the DNASep column (Transgenomic, Hillington, United Kingdom). The gradient was prepared by mixing buffer A [0.1 M triethylammonium acetate buffer (TEAA), pH 7.0] and buffer B [25% acetonitrile in 0.1 M TEAA]. Extension products were typically eluted using a linear gradient from 18% B to 38% B. RNA editing efficiency was calculated by comparing the area of the peak corresponding to the edited and nonedited extension products.

For the mouse study, heterozygous *Adar2* knockout mice (*Adar2*^+/−^ (n = 10) and the WT littermates (n = 10) were used for the RNA editing analysis by pyrosequencing [43]. Each group included five males and five females. Three brain areas (cerebral cortex, hippocampus, and cerebellum) were dissected from the brain of each mouse. RNA was extracted with TRIzol reagent (Invitrogen, Carlsbad, CA). In brief, after RT-PCR with a biotinylated primer, streptavidin-sepharose beads (GE Healthcare Life Sciences, Uppsala, Sweden) and the binding buffer (10 mM Tris–HCl, 1 mM EDTA, 2 M NaCl, 0.1% Tween 20 at pH 7.6) were mixed with the RT-PCR product. The reaction mixture was placed onto a MultiScreen-HV clear plate (Millipore, Billerica, MA). After applying the vacuum, the beads were treated with a denaturation solution (0.2 N NaOH). The beads were then suspended in annealing buffer (20 mM Tris-acetate, 2 mM Mg-acetate at pH 7.6) containing a sequencing primer. The template-sequencing primer mixture was transferred onto a PSQ96 Plate (Qiagen, Venlo, Netherlands). Sequencing reactions were performed with a PSQ96 SNP Reagent Kit (Qiagen) using PSQ96MA (Qiagen) according to the manufacturer’s instructions.

### Behavioral analyses

Behavioral analyses were performed at BMSRC (Akashi, Japan) with *Adar2*^+/−^ (n = 16) and the WT littermates (n = 16). All were males, aged 8–10 weeks at the initiation of the behavioral analyses. The analyses were performed in the following order: open-field test, prepulse inhibition (PPI) test, elevated plus maze test, Morris water maze test, passive avoidance learning test, active avoidance learning test, and forced swimming test.

### Open-field test

A transparent cubic box without a top (30 × 30 × 30 cm) was used. A 40-W white lamp provided room lighting, which was approximately 110 lux on the floor of the chamber. A fan attached to the upper part of the wall at one end of the chamber presented a masking noise of 45 dB. Two infrared beams were set on each wall 2 cm above the floor at intervals of 10 cm. The total number of successive interceptions of the two adjoining beams on each bank was scored as locomotion behavior. For the rearing analysis, 12 more infrared ray beams attached 4.5 cm above the floor at 2.5-cm intervals were used. The total number of vertical beam interceptions was scored as the rearing behavior. Each mouse was allowed to explore freely in the open-field area for 20 min.

### Startle response and PPI test

Using a transparent acrylic box (7 × 7 × 10 cm), the startle response was detected by an accelerometer (GH-313A, Keyence, Osaka, Japan) as vibration of the box. The acoustic startle pulses of a broadband burst (115 dB, 50 ms) and tone prepulse (85 dB, 30 ms) were presented by using a speaker located in front of the box. Prepulse using light (30 ms) was also applied by a light-emitting diode (LED); however, this was not used as data because it was found that the light prepulse did not attenuate the startle response.

At the beginning of the session, 40 startle pulses were presented to test for basal startle responsiveness and its habituation. The average values of eight blocks, consisting of five startle pulses each, were used for the statistical analysis. After that, three different types of trials were performed; that is, startle pulse alone (n = 12), startle pulse preceded by a tone prepulse (n = 12), and startle pulse preceded by a light prepulse (n = 12). Prepulses were presented 50, 100, or 200 ms before the startle pulse. In total, six types of prepulse (each n = 4) were applied. The mean interval averaged 25 s (15–45 s) throughout the session. The startle response was recorded for 200 ms with a sampling frequency of 1000 Hz. The PPI test was assessed by the ratio of the mean response of the trials with one type of prepulse (n = 4) divided by the mean response of the trials without prepulse (n = 12).

### Elevated plus maze test

The maze consisted of four arms (two open arms and two closed arms), 5 cm wide and 30 cm long with a gray acrylic floor, which met at a 10 × 10 cm center zone. The two closed arms had transparent walls (15 cm in height) on both sides, and the open arms had low walls (3 mm in height) on both sides. The apparatus was mounted 75 cm above the floor of the room. The room lighting was approximately 20 lux on the maze. A video camera was placed 80 cm above the maze. A fan generated a masking noise of 45 dB. The animal was placed gently onto the center of the maze and was allowed to explore the maze freely for 10 min. The number of entries into each arm and the time spent in the open arms were videotape recorded.

### Morris water maze test

A round pool, with a diameter of 95 cm and depth of 21.5 cm, was placed in the center of a 140 × 130 cm room. A platform with a diameter of 11 cm was set in one of the quadrants, 5 mm beneath the surface of black water maintained at 21 ± 1°C. On the first to fifth days, five trials per day were performed for the learning phase. Each mouse was released in one of the three quadrants of the pool without the platform, and the time to reach the platform was measured. If the mouse could not reach the platform within 60 s, the experimenter placed the mouse on the platform. On the sixth day, a probe test was performed to examine whether the mouse remembered the platform’s location. The mouse was released in the quadrant on the opposite side of the platform, and its behavior for 60 s was videotaped. The time the mouse stayed in the target quadrant where the platform had been placed and the immobility time were measured.

### Passive avoidance learning test

A mouse was placed in a box consisting of two rooms separated by a shutter; that is, light and dark compartments (each 10 × 10 cm). In the acquisition trial, the mouse was kept in the light compartment. Five seconds later, the door to the dark compartment was opened. When the mouse moved into the dark compartment the shutter was closed and 10 s later an electrical shock (160 V, 3 s) was delivered through the grid floor. Twenty-four hours later each mouse was placed again in the light compartment and the latency to enter the dark compartment was recorded up to a maximum of 180 s.

### Active avoidance learning test

The same apparatus used for passive avoidance learning was also used in the active avoidance learning test; however, there was no shutter between the light and dark compartments. The box was set in a soundproof chamber and illuminated by a 20-W white light set on the chamber. The ceiling of the dark room was made of black acryl board and the ceiling of the light room was transparent acryl board.

The training was performed for 3 days. On each day, one session consisting of 50 trials was performed. In each trial, a condition stimulus (CS) of a 1500-Hz sound (85 dB) was followed by an unconditioned stimulus (US) of a 140-V electrical shock. The US was given 5 s after initiation of the CS and continued until the mouse escaped to the other compartment. If the mouse did not move to the other compartment, the US lasted 15 s together with the CS. If the mouse moved within 5 s after the CS, the CS was stopped and no US was given. The time from the CS to the escape and the number of escapes were used to measure the learning performance.

### Forced swimming test

The animals were placed in a square pool 24 × 24 cm in size. The water temperature was maintained at 21°C. On the first day, the mice were left in the pool for 20 min and the mobility recorded during the first 5 min by the video camera was assessed. On the second day the animals were placed in the pool for 5 min and the immobility time over 5 min was recorded. The immobility was defined by two criteria: 1) no movement of the legs and the tail and 2) a completely stationary state in the pool or movement only by inertia from previous movement. The immobility was assessed from the video by three independent raters. The median value of the three raters was used for the analysis.

### Pharmacological experiments

Pharmacological experiments were performed at the RIKEN Brain Science Institute. For this analysis, *Adar2*^+/−^ mice (n = 34) and the WT littermates (n = 30) were analyzed. The laboratory was air-conditioned and the temperature and humidity were maintained at approximately 22–23°C and 50–55%, respectively. Food and water were freely available except during experimentation. All of the experiments were conducted in the light phase (from 9:00 a.m. to 6:00 p.m.), and the starting times of the experiments were kept constant.

For analysis of locomotor activity, an apparatus for the open-field test equipped with a small soundproof room (185 × 185 × 225 cm) was used. Each field was made of white plastic (50 × 50 × 40 cm) and illuminated by LEDs (70 lux at the center of the field). The behavior of the mouse was monitored by a charge-coupled device camera placed on the ceiling of the rack for the open fields. The distance traveled (cm) was analyzed every 5 min. Data were collected and analyzed using an Image J OF4 (O’Hara & Company, Ltd., Tokyo, Japan), which is a modified software based on the public domain U.S. National Institutes of Health (NIH) image program (developed at the NIH and at http://rsb.info.nih.gov./nih-image/).

Amphetamine was dissolved in an aqueous 0.9% NaCl solution (1 mg/mL). The stock solution was further diluted with the 0.9% NaCl solution to achieve a dose of 1.5 mg/kg in 200 μL for injection. The locomotor activity of mice was observed for 50 min before subcutaneous administration of the amphetamine solution. In the first experiment, the fold change of the activity over 50 min after the administration of amphetamine or saline compared to the activity before treatment was used as the index.

NBQX (2,3-dioxo-6-nitro-1,2,3,4- tetrahydrobenzo[f]quinoxaline-7-sulfonamide) was dissolved in a saline solution (0.9% NaCl in water) (100 mg/mL). This stock solution was further diluted with the saline solution to achieve an dose of 10 mg/kg in 200 μL for intraperitoneal injection after a 50-min adaptation period. Fifty minutes after NBQX dosing, amphetamine or saline was administered subcutaneously. The fold change of the activity from 50 min after amphetamine treatment compared to the locomotor activity during the middle 50 min after the NBQX treatment was used as an index. These two experiments used independent mice. The first experiment consisted of 11 WT and 10 *Adar2*^+/−^ mice and the second experiment consisted of 19 WT and 24 *Adar2*^+/−^ mice.

### Statistical analyses

For the statistical analysis, two-sample *t*-test, Pearson’s coefficient of correlation, one-way ANOVA, two-way ANOVA, and RMANOVA were used. For statistical analysis of behavioral battery, at first RMANOVA with factors of time and genotype was applied. When a statistically significant interaction was detected, further post hoc analysis by *t*-test was applied. The statistical analyses were performed using IBM SPSS Statistics version 20 (IBM Corporation, New York).

## Abbreviations

AMPA: 2-amino-3-(3-hydroxy-5-methyl-isoxazol-4-yl)-propanoic acid; ADAR2: Adenosine deaminase acting on RNA type 2; HTR2C: Serotonin 2C receptor; GRIA2: AMPA-type ionotropic glutamate receptor, subunit 2; GRIA3: AMPA-type ionotropic glutamate receptor, subunit 3; GRIA4: AMPA-type ionotropic glutamate receptor, subunit 4; GRIK1: kainate-type ionotropic glutamate receptor, subunit 1; GRIK2: kainate-type ionotropic glutamate receptor, subunit 2; PCR: Polymerase chain reaction.

## Competing interests

The authors declare no conflict of interest regarding this work.

## Authors’ contribution

MKS performed behavioral and pharmacological analysis. KI and MB performed gene expression, RNA editing and behavioral analyses. TK organized the project, analyzed the data and wrote the paper. All authors read and approved the final manuscript.

## Supplementary Material

Additional file 1: Figure S1 No significant effect of NBQX on locomotor activity in both WT and *Adar2*^+/−^ mice. Treatment with NBQX does not suppress a locomotor activity in both WT and *Adar2*^+/−^ mice. Click here for file

Additional file 2: Table S1 List of primers used in this study. Forward and reverse primer sets used in RNA editing analysis and RT-PCR for human and mouse samples. Click here for file
