# Supplementary material for: A role of ADAR2 and RNA editing of glutamate receptors in mood disorders and schizophrenia
Source: Mol Brain. 2014 Jan 21;7:5. doi: 10.1186/1756-6606-7-5 (PMC3902024; doi:10.1186/1756-6606-7-5)
Supplement: Additional file 1: Figure S1 — No significant effect of NBQX on locomotor activity in both WT and Adar2+/− mice. Treatment with NBQX does not suppress a locomotor activity in both WT and Adar2+/− mice. [file 1756-6606-7-5-S1.pdf]

Additional Figure 1

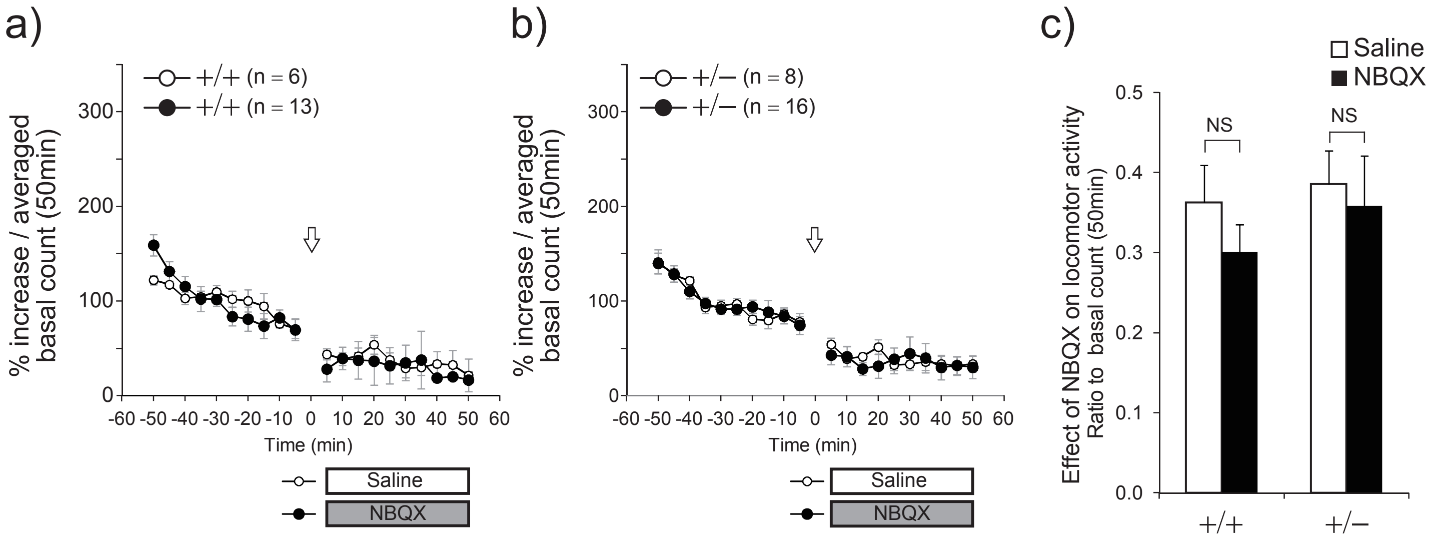

No significant effect of NBQX on locomotor activity in both WT and *Adar2*$^{+/-}$ mice.

a, b) Locomotor activity gradually declined in two groups with treatment of saline or NBQX in both the WT (a) and the *Adar2*$^{+/-}$ mice (b). c) The vertical axis indicates the fold change of the averaged activity level during 50 min after the administration of NBQX to the averaged activity during the 50 min before the treatment. There was no significant difference of activity level between the saline-injected and the NBQX-injected groups both in the WT mice (the saline-injected group, open column [0.36 ± 0.05]; the NBQX-injected group, closed column [0.30 ± 0.03]; $P = 0.71$, Student' s *t*-test) and the *Adar2*$^{+/-}$ mice (the saline-injected group, open column [0.39 ± 0.04]; the NBQX-injected group, closed column [0.36 ± 0.06]; $P = 0.27$, Student' s *t*-test). This suggests that the NBQX treatment does not suppress a locomotor activity.

*For this figure, the data sets were used from figure 4c, d. To avoid a repeated use of the data, it was not shown in the main manuscript.
